# Impact of COVID-19 on Research in Durham University Business School

**DOI:** 10.1177/21582440231181314

**Published:** 2023-06-16

**Authors:** Richard Harris

**Affiliations:** 1 Durham University Business School, UK

**Keywords:** EDI, COVID-19, research impact, gender, ethnicity

## Abstract

Statistically robust evidence that the pandemic (C19) has had an adverse impact on academic research carried out in Universities is limited. The new results presented are based on a survey of Business School academics who were entered into the Research Excellence Framework (REF) 2021 assessment of research quality, confirming that C19 had a major effect during the March to September 2020 period on research activities. In terms of which sub-groups of staff have been most affected, the largest negative effects are associated with those (almost all female) staff who took paternity/maternity leave during the 7-year REF period; followed by female staff, those (mid-career researchers) in the Associate Professor grade, then staff classified as “other white ethnic” (as opposed to White British). The implications of this for equality, diversity, and inclusion are likely to be significant, as is discussed when looking at what universities might do to overcome the negative impacts of C19.

## Introduction

There is limited, but a growing body of evidence, some of which is reviewed below, that the pandemic (hereafter denoted C19) has had an adverse impact on academic research carried out in Universities; however, there is less “hard” statistically backed confirmation that shows what has been happening. The expectation with some verification provided is that female academics have been most disadvantaged, having less time to do research related activities. But even for this sub-group, evidence is limited both in terms of the range of research activities covered and the extent to which females have been adversely impacted, when other factors are also taken into account.

Whether C19 has had a greater presumed negative impact on certain sub-groups—such as females, different ethnic groups, and early- or mid-career academics—is important in the wider context of equality, diversity, and inclusion (EDI), which the university sector has been pursuing now for a number of years. It is recognized that there exists an imbalance in the representation of these sub-groups at higher grades in universities, and there are difficulties in ensuring recruitment and promotions adhere to an approach based on meritocracy (Nielsen, 2016) and the associated need for greater equity (i.e., not just equal opportunity and access but also equal outcomes—cf. Angervall & Beach, 2020; Barrow & Grant, 2019). As Scott (2020) notes, “diversity is going to a party and inclusion is being asked to dance… (and also) choosing the music” (pp. 179–180). Consequently, there is a concern that C19 might have reduced any gains achieved by EDI, or if such gains are contestable—see Beattie and Johnson (2012), Scott (2020), Ooms et al. (2019), and Nielsen (2016)—then C19 has reinforced the limits to meritocracy in the system. Going forward, unless universities take steps to mitigate the impact of the pandemic, it is likely that imbalances in research across EDI sub-groups will almost invariably increase in the next few years.

The results presented here are based on a survey of Durham Business School academics entered into the Research Excellence Framework (REF) 2021 assessment of research quality. Significant differences in responses to C19 are found for females versus males, there is some association with ethnicity, but the largest disadvantaged sub-group are those overwhelmingly female staff who have taken maternity leave during the REF period, indicating the presence of young children in the home environment. Indeed, when a range of potential covariates (including gender, maternity/paternity leave, department, and academic grade) are considered, the direct importance of gender often becomes statistically insignificant as it is the presence of young children that is often most associated with the impact of C19 on research.

The rest of this article is organized as follows: the next section overviews recent work in this area and is followed by details about the survey conducted with an overview of the main results obtained. There is then a deeper look at the impact of C19 on research based on multivariate analysis, that takes into account a range of staff characteristics when analyzing the extent to which the “stay-at-home” policy of Durham University, and associated inability to access on-site facilities, as well as a national lockdown (March 23rd until late June followed by some easing of restrictions), impacted on research. Finally, there is a summary and conclusions.

## Evidence of the Impact of C19 on Research

Early evidence of the impact of C19 on research has mostly relied on a comparison of pre- and post-pandemic submissions of working papers, pre-prints, and in some instances limited information on submission rates to journals (see Oleschuk, 2020; Peters, 2020; Viglioni, 2020, for detailed reviews). Thus, Frederickson (2020) compared pre-prints registered on arXIV and bioRxiv for the period 15th March to 15th April 2020, compared to the same period in 2019, finding a relative reduction in sole female authorship submission rates. As the author acknowledges, many of these preprints was based on research completed months before the pandemic, assignment of gender is not perfectly predictive using the approach taken in this type of work, and domestic circumstances (e.g., whether there were children in the household), and other personal characteristics are unknown. Vincent-Lamarre et al. (2020) undertook a similar exercise based on the medRxiv, bioRxiv, and arXiv preprint repositories, for March to April 2020, with similar conclusions, including additional information that females were less likely to be involved in C19 studies when compared to males. Shurchkov (2020) also carried out a comparable study, producing analogous results, based on a database that tracks new publications primarily for economists covering major working papers series and preprint repositories (e.g., arXiv Economics; see also Inno et al., 2020) for Italian astronomy and astrophysics researchers). The impact of C19 on economists is confirmed by Amano-Patiño et al. (2020) using similar working paper series, although they found that the relative number of female authors in non-pandemic research was stable and the main downturn was in female authors working on C19 research topics (especially mid-career female economists). Cui et al. (2020) use preprint submissions to the SSRN database that covers 18 social science disciplines, finding females during 10 weeks of lockdown in the US were relatively less likely by over 13% to be contributing compared to males. The largest study of this type comparing gender impacts is Squazzoni et al. (2020), who had access to over 5 million authors and referees from over 2,300 Elsevier journals for February 2018 to May 2020. They found that during the first wave of the pandemic women submitted fewer papers and were less likely to act as reviewers on papers submitted, when compared to men. These differentials were apparent across a wide spectrum of academic subjects (only omitting Arts and Humanities, which were generally not covered in the Elsevier journals list). Note, little statistical analysis was undertaken in the studies so far covered, beyond mostly graphical comparisons, mainly because the studies concerned did not have access to the underlying characteristics of those undertaking the research. Note others have done similar work limited to specific subject areas—for example, Gabster et al. (2020) for medicine. Others (e.g., Flaherty, 2020) refer to evidence from three journal editors in April 2020 where the editors note that submission by females appeared to be lower (Fazackerley, 2020; Fuchs-Schündeln, 2020; Wiegand et al., 2020, provide further evidence). The main exception was Cui et al. (2020) who estimated a regression model which included gender and a “lockdown dummy” as determinants, but no other control variables.

Other studies have considered the extent to which female academic experts have been interviewed during the pandemic (BMJ, 2020; Lines, 2020), but much of this evidence is anecdotal. In addition, information from real-time surveys suggest women spend relatively more time on pandemic-era childcare and home schooling (Adams-Prassl, et al., 2020; see also, e.g., Boncori, 2020; Jedd et al., 2020; Shollen et al., 2009). The closest studies to that undertaken here are Myers et al. (2020) and Deryugina et al. (2021). The first is based on data collected in April 2020 from 4,535 academics in the US and Europe which shows how time spent on academic duties (covering research, teaching fundraising, and other tasks) was adversely affected, most especially research and fundraising. With respect to research time, they found the largest relative falls were associated with certain subject areas such as science-based requiring laboratory experiments, being female and having young children present in the household (note, being both female and having young children provided overall the largest negative impact). They were able to conclude that “… when everything else is held constant, gender and young dependents play a major role” (Myers et. al., op. cit., p. 881). They also noted that their survey response rate was only 1.6% comprising a self-selected sample, likely over-representing those who felt strongly about their experiences.

Staniscuaski et al. (2021) also undertook survey work collecting data for over 3,300 Brazilian academics during 22nd April to 25th May 2020. The sample was obtained using a snowball sampling technique with over 68% of the respondents being females. The main information collected was whether papers were submitted as planned. Besides issues of possible selection bias, the short period for collecting information linked to paper submission rates is also problematic. The results, however, confirmed female academics (especially those with children) were most (negatively) affected by C19. Krukowski et al. (2021) also used a snowballing sample to obtain 284 responses (nearly 68% of whom were females) from Science, Technology, Engineering, and Maths (STEM) faculty in the U.S. comparing mid-January to mid-March 2020 with the same period in 2019. Only univariate tests were conducted of hypotheses with similar results to other studies showing female academics (especially with young children) experience the largest negative impact on research.

Deryugina et al. (2021) conducted an even larger survey of academics than Myers et al. (2020), including outside the US and Europe and with nearly 28,000 responses (although this was still only around a 2.2% response rate of usable returns). This larger study considered time allocated to research, other work, and then a range of other commitments such as commuting, childcare, housework, and sleep. They regressed time use on gender and the presence of children, having also controlled for when the researcher obtained their PhD, race, and ethnicity. The key univariate results were that female academics experienced larger falls in research time and commuting coupled with greater time allocated to childcare and housework and marginally less time for sleep, when compared to males. The main multivariate result was that the pandemic had adversely affected the research productivity of female academics with especially young children. As will be seen, these results are similar to those presented below although in the present study there is more confidence that the survey results are statistically unbiased, and the study covers a wider range of variables associated with research than just time allocated.

## Methods

The entire population of 132 Category A staff entered into REF 2021 were surveyed in early October 2020, that is, those with at least a 0.2 fulltime equivalent (FTE) contract at 31^st^ July 2020. Respondents were asked to rate the impact of C19 from the end of March 2020 to September 2020 on their ability to undertake research activities. The survey instrument is available in an online Supplemental appendix, with certain questions (such as gender, ethnicity—based on ONS definitions—and reasons for career breaks during the REF period of January 2014 to December 2020) randomized in terms of the options available for selecting, in order not to imply any weighting to any particular answer. Non-respondents were sent a follow-up email 5 days after the initial email went out; and there was a final reminder 10 days after the initial email. The final response rate was 60.6%. Table A1 in the appendix presents the profile of those who responded against the profile of the population surveyed; the correlation between the profiles of the respondents and the population is 0.99 suggesting the sample is representative.

Six key questions (see Table 1) relating to the impact of C19 asked staff to indicate on a sliding scale from −100 (unable to undertake any substantive research) to +100 (devoted 100%+ more time to research) what had been the effect of C19 on the following: (quality) research time, production of journal articles, applying for research grants, and impact and engagement activities.

**Table 1.** Average Response to Impact of C19 on Research, March to September 2020.^a^

	Department			Position			Gender		ECR	
	Accounting	Economics and Finance	Management and Marketing	Assistant Professor	Associate Professor	Professor	Male	Female	No	Yes
Since the end of March 2020 what has been the impact of/effect:										
Working from home on the amount of time you have devoted to maintaining your research agenda	7.8**	−25.8*	−14.9	−16.2	−27.0	−10.3	−9.6	−33.9**	−17.7	−16.0
Working from home on the quality of time you have devoted to maintaining your research agenda	10.0***	−30.3*	−21.5	−21.0	−30.4	−15.9	−15.4	−36.2**	−23.0	−16.0
On your production of papers (about to be) submitted to journals compared to what you would likely have achieved (or had planned to achieve) in the absence of C19?	6.7**	−26.8*	−18.8	−16.7	−30.4	−12.8	−11.3	−37.3***	−21.1	−10.0
On your production of papers (about to be) submitted to 4* journals compared to what you would likely have achieved (or had planned to achieve) in the absence of C19?	−6.7***	−28.4	−22.1	−21.9	−33.0	−16.3	−15.2	−40.4***	−23.6	−22.0
On your applying for research grants (about to be) submitted to funding bodies compared to what you would likely have achieved (or had planned to achieve) in the absence of C19?	−14.4	−31.3	−20.0	−26.2	−43.7**	−7.8***	−18.9	−36.9*	−25.6	−19.0
On your undertaking impact and engagement activities compared to what you would likely have achieved (or had planned to achieve) in the absence of C19?	−20.0	−31.1	−41.2	−38.6	−43.0	−23.4*	−24.8	−53.1***	−33.0	−41.0
***Average***	***0.1*** **	***−26.5***	***−21.4***	***−21.5***	***−31.2*** *	***−13.0*** *	***−13.4***	***−38.0*** ***	***−21.6***	***−19.9***

a Based on a sliding scale from −100 (unable to undertake any substantive research) to +100 (devoted 100%+ more time to research).

*/**/*** Significantly different at 10%/5%/1% level based on *t*-test.

**Table:** 

	White British		Other White ethnic		Took parental leave in REF period		Had time off from research in last 7 years		Total
	No	Yes	No	Yes	No	Yes	No	Yes	
Since the end of March 2020 what has been the impact of/effect									
Working from home on the amount of time you have devoted to maintaining your research agenda	−22.0	−4.0*	−11.9	−25.9	−12.5	−56.7***	−17.2	−22.0	−17.5
Working from home on the quality of time you have devoted to maintaining your research agenda	−26.5	−9.0*	−19.4	−26.3	−18.3	−52.2***	−21.7	−28.0	−22.1
On your production of papers (about to be) submitted to journals compared to what you would likely have achieved (or had planned to achieve) in the absence of C19?	−23.3	−9.0	−16.3	−25.0	−15.8	−51.1***	−19.7	−20.0	−19.8
On your production of papers (about to be) submitted to 4* journals compared to what you would likely have achieved (or had planned to achieve) in the absence of C19?	−27.8	−10.0**	−20.6	−27.5	−20.9	−43.3*	−23.5	−22.0	−23.4
On your applying for research grants (about to be) submitted to funding bodies compared to what you would likely have achieved (or had planned to achieve) in the absence of C19?	−32.2	−2.5***	−18.8	−33.8	−20.0	−62.2***	−23.7	−40.0	−24.8
On your undertaking impact and engagement activities compared to what you would likely have achieved (or had planned to achieve) in the absence of C19?	−39.3	−18.0**	−25.8	−46.3**	−32.5	−45.6	−34.7	−24.0	−34.0
***Average***	***−25.7***	***−8.4*** **	***−17.6***	***−27.1***	***−17.6***	***−51.3*** ***	***−21.2***	***−23.8***	***−21.4***

*/**/*** significantly different at 10%/5%/1% level based on *t*-test.

It is important to note that during the period covered by the survey, most all staff were working from home; only a very small number around 3% had returned to their offices by late July to conduct “essential” research projects. Durham University’s policy at this time was that staff should work from home unless they could not conduct essential research without access to their office (or laboratory in the case of scientists). In the Business School, for health and safety reasons, essential research was specifically defined to make office working the exception rather than the norm. Staff maintained access to databases and journals using their home computers, and extensive use was made of Zoom and Microsoft Teams to maintain links with other colleagues and collaborators. However, there were clearly domestic and psychological limitations imposed by “lockdown” that impacted on research, and during June to September 2020 there was also the need to prepare for “blended” teaching in term 1 of the 2020 to 2021 academic year, a “new” form of teaching for most academics and which involved a significant extra level of workload preparation. For example, information collected by the University on how academics allocated their time shows that for Business School staff the overall percentage of time allocated to teaching/teaching support increased from 31% in 2018/2019, to 36% in 2019/2020 and 43% in 2020/2021.

Against this background, Table 1 (final column) shows that the average response across all staff was that C19 had had a major effect, lowering the amount of time devoted to research (−17.5%), lowering quality time for research by even more (−22.1%), and reducing the ability to produce papers for journals by around 20% to 24% depending on the quality of the article(s) prepared. Applications for externally funded grants were judged to be nearly 25% lower, while impact and engagement activities, including attendance at conferences, workshops, talks, providing expert advice, etc., were down by 34%. Table 1 also shows there were significant variations across the different sub-groups of respondents. In general (cf. the final row), staff in Accounting were more optimistic compared to those in the other two departments (especially Economics and Finance). Associate Professors who are mostly mid-career staff, stated they were particularly disadvantaged during the period up to October 2020. In most all areas covered in Table 1, female staff provide a more negative view on the impact of the pandemic compared to men, especially those that took maternity leave during the REF period, indicating the presence of small children at home during the pandemic. All the differences based on gender, and most based on whether paternity/maternity leave occurred, are statistically significant (based on a simple *t*-test that the difference was significantly different to 0). There is also some indication in Table 1 that those staff describing themselves as White British (see Table A1 for the full range of definitions used) experienced a lower negative impact compared to all other staff, for example, the amount of (quality) time devoted to research; producing paper for 4* journals; applying for research grants; and impact and engagement activities.

## Discussion of Multivariate Results

The results presented in Table 1 only make univariate comparisons such as females versus males. The average values presented do not consider the “mix” of different characteristics that make up each sub-group (e.g., while females accounted for 30.3% of the population of category A research staff, only 18.2% of professors were female; in terms of the sample of 80 who completed the survey, 18.8% of professors were female). To obtain a more in-depth picture of the relationship between different sub-groups of staff and the impact of C19, the covariation between different characteristics and impact needs to be included. Therefore regression analysis was used, to show the partial association/correlation between the impact of C19 and a particular characteristic, having “controlled for” the influence of all other characteristics (all other variables) in the regression model. Thus, the following model was estimated using ordinary least squares (OLS) regression:

(1) $$\begin{array}{c} Y_{i}=b_{0}+b_{1}\mathrm{Femal}e_{i}+b_{2}\mathrm{Young}\;\mathrm{chil}d_{i}+b_{3}\mathrm{Accountin}g_{i}\\ +b_{4}\mathrm{Marketing}\;\&\;\mathrm{Manageme}t_{i}+b_{5}\mathrm{White}\;\mathrm{Britis}h_{i}\\ +b_{6}\mathrm{Other}\;\mathrm{white}\;\mathrm{ethni}c_{i}+b_{7}\mathrm{EC}R_{i}+b_{8}\mathrm{Professo}r_{i}\\ +b_{9}\mathrm{Associate}\;\mathrm{professo}r_{i}+b_{10}\mathrm{Post}-\mathrm{ECR}\;\mathrm{years}\;\mathrm{as}\;\mathrm{researche}r_{i}\;\\ +b_{11}\mathrm{Had}\;\mathrm{time}\;\mathrm{off}\;\mathrm{in}\;\mathrm{REF}\;\mathrm{perio}d_{i}+\varepsilon_{i} \end{array}$$

The dependent variable in equation (1) comprises the answer (ranging from −100 to +100) to each of the questions listed in Table 1 for each respondent *i*; all the other (right-hand side) discrete (0/1) variables in the model take on a value of 1 if the characteristic is “true,” for example, the respondent is female. Only “post-ECR years as a researcher” is a continuous variable measured in number of years. Finally, $\varepsilon_{i}$ is a “residual” terms that picks up all other unmeasured influences on the dependent variable. Strictly speaking, $\varepsilon_{i}$ is presumed to be a normally-distributed random variable with zero mean, constant variance. When estimating equation (1), some tests as to whether this OLS residual met this criterion were performed (e.g., for heteroscedasticity, normality, and the Ramsey RESET test for omitted variables) and generally the results were favorable. However, they are not reported here, as the use of OLS regression (estimating equation (1)) provides an indication of the main variables “explaining” the responses obtained—that is, more a test of statistically significant associations—rather than any attempt to estimate a causal model (which would require more data, and significantly more sophisticated techniques to be used).

**Figure 1.**
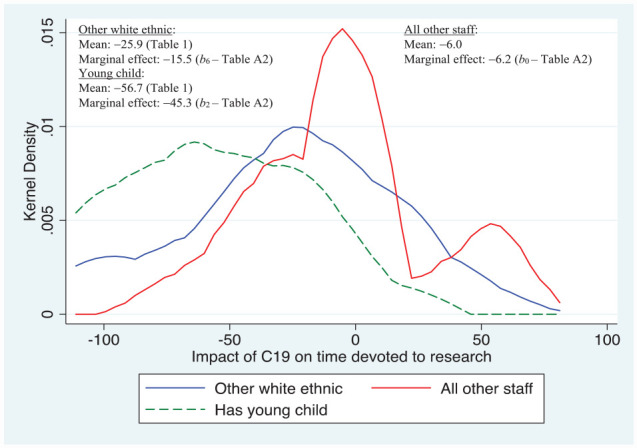
Major determinants of impact of C19 on time devoted to research. *Source*. Author’s own survey (see Supplemental Appendix).

Note, stepwise regression techniques were used to produce the preferred set of results reported in Table A2 in the appendix; these are reported here in the main text using a set of diagrams that concentrate on those significant characteristics associated with C19 that had the largest impact on research activities, that is, only statistically significant variables obtained when estimating equation (1) are included in the diagrams. Figure 1 shows the distribution of responses for the question relating to the impact on time devoted to research during March to September 2020. Clearly, the density of responses for those that took maternity/paternity leave are to the left of those in the other white ethnic sub-group (defined in Table A1), with the latter to the left of all other staff comprising those not in the other white ethnic or young child sub-groups, indicating larger negative impacts on research time for those with younger children, followed by the other white ethnic sub-group.

Compared to the baseline sub-group of all other staff, the marginal effect (i.e., the *ceteris paribus* association, $\partial y_{i}/\partial x_{i},$ between time devoted to research and the relevant sub-group) for those staff having young children equated to just over 45% lower research time. This is some 39 percentage points lower than the marginal effect for all other staff, while the difference in the overall means of both variables is nearly 51 percentage points, indicating that when other covariates are “controlled for” that are also associated with lowering research time, isolating the “young child” effect itself is not as large as the difference in “raw” mean scores (in fact, Table A2 indicates that the marginal effect for the baseline sub-group (of −6.2) is not statistically different to 0; with *b*_0_ set to 0, the difference in marginal effects would be just over 45 percentage points, closer to the difference in “raw” means.). The other sub-group to experience statistically significant lower research time, having controlled for other influences, comprises those classified as “other white”; their marginal effect was nearly 10 percentage points lower than for the baseline sub-group of all other staff and the difference in “raw means” was nearly 20 percentage points.

**Figure 2.**
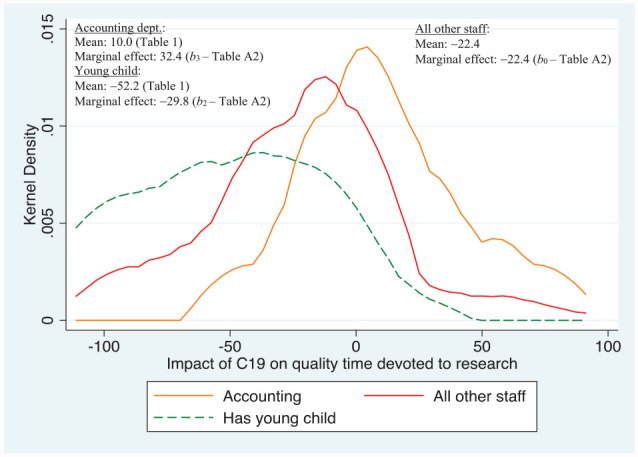
Major determinants of impact of C19 on *quality* time devoted to research. *Source*. Author’s own survey (see Supplemental Appendix).

Figure 2 shows the distribution of responses relating to the impact of C19 on quality research time, which as Table 1 shows was harder hit compared to general research time; the pattern is also more bi-modal. That is, compared to responses about research time in general (Figure 1), the other white ethnic sub-group is replaced by staff belonging to the Accounting Department who had a response density significantly to the *right* of other staff. Thus staff in Accounting overall were able to devote relatively much more time to quality research compared to others, with a marginal effect of +32.4%, which is nearly 55 percentage points higher than the baseline of all other staff, that is, those in other departments that also did not take maternity/paternity leave. Those with young children have a marginal effect of nearly −30%, which is over 7 percentage points lower than for all other staff comprising the benchmark sub-group.

**Figure 3.**
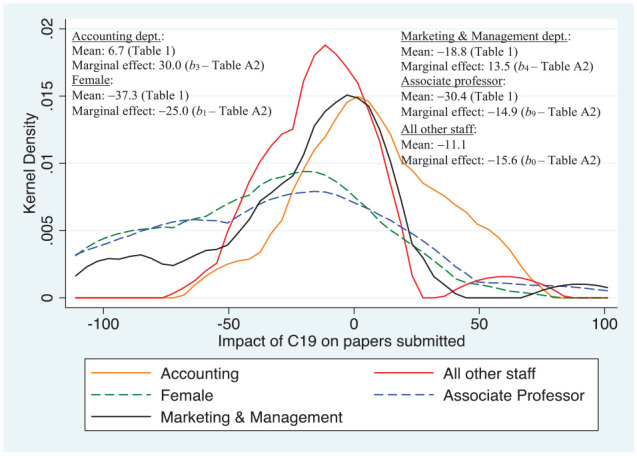
Major determinants of impact of C19 on papers prepared and/or submitted. *Source*. Author’s own survey (see Supplemental Appendix).

As to the production of papers about to be submitted to journals compared to what would likely have been achieved or planned in the absence of C19, Figure 3 shows that in terms of the marginal effect females were most disadvantaged; some 9 percentage points lower than for the baseline sub-group. Associate professors were next in terms of their negative marginal effect—much worse off than the two department sub-groups identified, but little different to the marginal effect of the baseline sub-group; when “raw means” are compared, those in the associate professor sub-group reported much lower submission rates. In comparison, staff in Marketing & Management had a marginal effect of 13.5, some 29 percentage points higher than the baseline sub-group, and nearly 39 percentage points higher than the female sub-group. However, staff in Accounting did even better: their marginal effect was 30%, some 55 percentage points higher than those for females.

**Figure 4.**
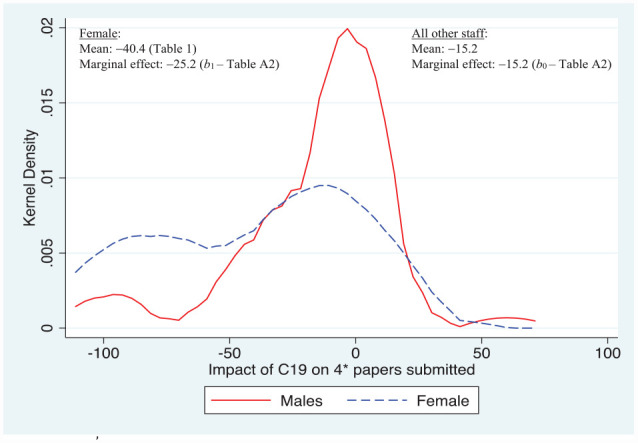
Major determinants of impact of C19 on 4* papers prepared and/or submitted. *Source*. Author’s own survey (see Supplemental Appendix).

While Figures 2 and 3 indicate that staff in the Accounting Department did relatively much better in terms of avoiding the negative impact of C19 on quality research time and producing papers for journals, Figure 4 suggests that this advantage was not carried over into producing papers for the highest ranked journals. This may, in part, reflect the number of accounting journals available to publish in at this level. Only the female sub-group had a statistically significant marginal effect, which was 10 percentage points below the baseline sub-group comprising in this instance males.

**Figure 5.**
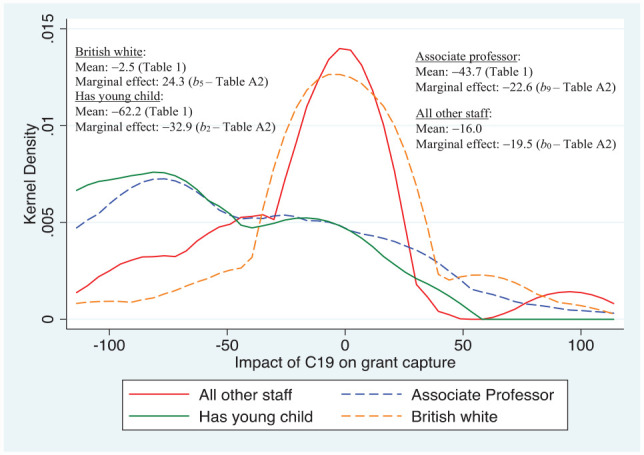
Major determinants of impact of C19 on research grant capture. *Source*: Author’s own survey (see Supplemental Appendix).

Table 1 indicates that overall applying for research grants was on average −24.8% lower than it likely would have been in the absence of C19. Figure 5 shows those sub-groups that were particularly vulnerable were, *ceteris paribus*, staff with young children that took maternity/paternity leave during the REF period, and Associate Professors. The marginal effects for those two sub-groups are over 13 and 3 percentage points lower than that of the baseline sub-group, which itself experienced a marginal effect of nearly −20%. In contrast, staff classified as British white had a marginal effect that was nearly 44 percentage points higher than the “all other staff” baseline sub-group, and the “raw mean” for this sub-group was over 22% higher than staff in general, and over 13% higher compared to the “all other staff” sub-group in Figure 5, which excludes the other sub-groups depicted in the diagram.

**Figure 6.**
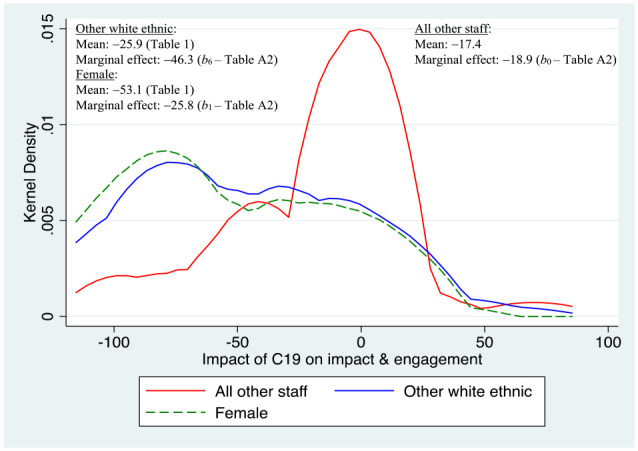
Major determinants of impact of C19 on impact and engagement. *Source*: Author’s own survey (see Supplemental Appendix).

In terms of impact and engagement (Figure 6), the two sub-groups to experience the worst marginal effects were females and those belonging to “other white ethnic.” While the “raw mean” for females was lower, by nearly 36 percentage points compared to all other staff in the diagram, the marginal effect controlling for “all other effects” was over 27 percentage points lower for the other white ethnic sub-group relative to the baseline (compared to just over 8 percentage points lower for females).

Overall, the results presented in Table A2 and summarized in Figures 1 to 6 show that females and especially those with young children in the household experienced, ceteris paribus, the largest negative impacts from the effect of C19 on research. Research was also adversely affected in the “other white ethnic” and Associate professor sub-groups, although not to the same extent. This confirms the “raw means” data summarized in Table 1.

## Summary and Conclusions

UK universities are becoming increasingly engaged in tackling equality, diversity, and inclusion (EDI) issues, given that historically they have tended to over-recruit and promote (older) white British males. Before the pandemic, efforts were being made to increase the proportion of especially female staff in higher academic grades to mirror more closely the gender balance already in place, as well as recruit generally more females into academia. But while universities inevitably argue that recruitment, promotion and more generally resources for research operate in a system based on meritocracy, there are clearly outstanding concerns about the extent to which the system works given the under-representation of certain categories in more senior positions. Studies like those of Ooms et al. (2019) show that the main issue seems to be linked to progression to higher grades rather than recruitment to more junior research posts. Cheng (2020) looked at the career path of biological science academics, finding no gender gap in tenure track rates among individuals who had never had children (and among individuals before they had children), but a significant negative impact after the birth of the first child. Huang et al. (2020) come to a similar conclusion when they covered 13 disciplines, 83 countries, and the publishing career of academics between 1955 and 2010: they found “… we find that men and women publish a comparable number of papers per year and have equivalent career-wise impact for the same total number of publications… this suggests the productivity and impact of gender differences are explained by different publishing career lengths and dropout rates” (p. 4609). Hence the growth in importance of achieving Athena Swan certification is an indication of this recent awareness of EDI issues.

It seems likely, based on the results presented here, that C19 will have had a significant negative impact on such efforts, since females (and especially those with young children) have seen their research efforts curtailed to a greater extent than males. Given the emphasis on quality publications and securing grant income when recruiting and promoting (that is, measures of esteem, which some argue are more easily acquired by male academics due to a variety of factors of which the “alpha male” characteristic is often emphasized—for example, Coate & Howson, 2016), and the general tendency towards a greater culture of audit of academic performance in recent decades (Welch, 2016, p. 511, argues that the effects of this “… have significantly distorted academic mission, favoring research published in highly ranked international journals… enhancing gender differences in the profession”), C19 will likely have reduced (both now and going forward) the ability of females to increase their presence in the higher (paid) ranks of academia.

Therefore, the main conclusion from this study is that unless universities take steps to mitigate the impact of the pandemic, gender imbalance will almost invariably increase in the next few years. But how universities should respond presents a major challenge at all levels of such institutions (see, e.g., Malisch et al., 2020, who put forward the need for radical strategic action plans to combat the impact of C19 on minorities). The reasons often given as to why females (and ethnic minority sub-groups) are under-represented (particularly in senior research grades) include: (unconscious) bias in applying terms like “prestige,”“esteem,” and “excellence” is apparent when measuring research outcomes (Coate & Howson, 2016) in part due to “…‘male closeness’ and the ‘homosocial reproduction’ of gendered power relations” (Nielsen, 2016); such staff have insufficient mentoring, a lack of senior patronage, a paucity of supportive networks, inadequate socialization into academia, and this is explained in terms of patriarchy, male dominated norms and practices, women’s reticence to put themselves forward, and role conflict when prioritizing personal rather than professional lives. Subbaye and Vithal (2017) provide a useful review of these factors; Oleschuk (2020, p. 503) also overviews existing research that shows the performance of females is not reflective of differences in merit; and on the issue of whether periods of maternity leave truncate career progression, the evidence put forward by Coate and Howson (2016) was that “… most of the female respondents to this survey did not have children while most of the male respondents did… clearly the organisational culture and societal expectations are mainly to blame here … the idea that women’s childcare responsibilities hold them back is a convenient way for the institution to avoid doing much about gender inequality” (p. 582). Whether all these factors can be applied to the direct impact of C19 having a greater negative impact on females is less straightforward (other than time allocated to personal versus professional activities), but they will of course be important going forward in countering the longer-run impact of female staff likely falling further behind males in terms of research outcomes.

In the more immediate period when C19 is still having a direct impact, and as universities adjust towards a new “norm” (e.g., or “blended” teaching, undertaking research with social distancing protocols in place, how resources—including buildings and libraries—are allocated, etc.), there is also the need to take account of other relevant factors that are put forward as restraints affecting women relatively more than men. These include females experiencing more stress and suffering more from illness due to heavy workloads; the greater impact of balancing family responsibilities brought about by C19 restrictions; and “… for many women, this combination of public and private work creates a stressful situation, which is most probably related to understandings of femininity” (Angervall & Beach, 2020, p. 348). Having in place relevant HR responses to combat these problems is needed, alongside ensuring that the recruitment, progression, and promotion system does not extenuate the wider impacts of C19 on those whose research has been more seriously constrained. Overall, as Scott (2020,) argues, is the need for “… those who are developing and implementing (EDI) policies to do so in a way that challenges them to better understand the scale and nature of the problems, to do so in a way that is fully engaged with those who experience inequalities, and which develops a dialogic approach to address norms, practices and cultures. a reflexive approach” (p. 176). Perhaps, following the discussion in Scott (2020) and based on the Irish response to EDI, there is a need for short-run policies such as the adoption of gender quotas in promotions processes where the percentage of women promoted should at least equal the percentage of women at the grade below. Or course, this approach to fixing “… a dysfunctional system that disadvantages women” comes up against the argument that “… perceives preferential treatment as a threat to the stability of a well-functioning, objective, promotion system, where only the ‘best’ and ‘brightest’ succeed” (Nielsen, 2016, p. 386).

## Supplemental Material

sj-docx-1-sgo-10.1177_21582440231181314 – Supplemental material for Impact of COVID-19 on Research in Durham University Business School Click here for additional data file. Supplemental material, sj-docx-1-sgo-10.1177_21582440231181314 for Impact of COVID-19 on Research in Durham University Business School by Richard Harris in SAGE Open

## Appendix

**Table A1.** Sample Compared to Population From COVID-19 October 2020 Survey, Durham University Business School^a^.

	C19 survey^b^	Population^b^
Accounting Department	11.3	9.8
Economics and Finance Department	47.5	47.7
Management and Marketing Department	41.2	41.7
Assistant Professor	26.3	30.3
Associate Professor	33.8	28.0
Professor	40.0	41.7
Male	67.5	69.7
Female	32.5	30.3
Early career researcher (ECR)^c^	12.5	14.4
White British^d^	25.0	21.2
Other white ethnic^e^	40.0	40.2
Took paternity/maternity leave in REF period	11.3	6.8
Had time off in REF period	6.3	na
N (sample size)	80	132

*Note*. Figures are percentages.

a From the population of 132 Category A staff, 60.6% completed the C19 survey; 15.9% completed the EDI part but not the C19 survey; 23.5% did not complete either part.

b Pearson correlation between two columns is 0.99.

c Defined using the definition adopted by REF 2021 (see Q4 in the survey instrument available online for further details).

d Those classifying themselves as White English/Welsh/Scottish/Northern Irish/British (see Q3 in the survey instrument available online for further details).

e Those classifying themselves as one of the following: White Irish; White Gypsy; or Irish Traveler; Any other white background.

**Table A2.** OLS (Stepwise) Regression of Respondents Evaluation of the Impact of C19 on Research During March to September 2020 (see equation (1)).

	Since the end of March 2020 impact of/effect of C19 on:						
	Time devoted to research (full model)^a^	Time devoted to research	Quality time devoted to research	Production of papers	Production of 4* papers	Grant capture	Impact and engagement
Female (*b*_1_)	−15.7	—	—	−25.0***	−25.2***	—	−25.8***
Young child (*b*_2_)	−36.3**	−45.3***	−29.8**	—	—	−32.9**	—
Accounting Department (*b*_3_)	23.7	—	32.4**	30.0**	—	—	—
Management and Marketing Department (*b*_4_)	12.8	—	—	13.5*	—	—	—
White British (*b*_5_)	−0.1	—	—	—	—	24.3**	—
Other white ethnic (*b*_6_)	−12.7	−15.5*	—	—	—	—	−16.9*
ECR (*b*_7_)	−21.1	—	—	—	—	—	—
Professor (*b*_8_)	−12.4	—	—	—	—	—	—
Associate professor (*b*_9_)	−21.3	—	—	−14.9*	—	−22.6**	—
Post-ECR years as researcher (*b*_10_)	−0.1	—	—	—	—	—	—
Had time off in REF period (*b*_11_)	−14.4	—	—	—	—	—	—
Constant (*b*_0_)	6.5	−6.2	−22.4***	−15.6**	−15.2***	−19.5***	−18.9***
Observations	80	80	80	80	80	80	80
Adj-*R*^2^	.134	.139	.125	.175	.102	.170	.124

a Including all the (insignificant) regressors included in equation (1) has a small impact on the results, in terms of the parameter values obtained, although there is a tendency to lower the statistical significance attached to these parameters (hence the main reason for dropping such “nuisance” regressors). The first two columns of results provide an indication of the differences obtained from the “full” and parsimonious regression results.

*** *p* < .01. ***p* < .05. **p* < .1.
