# Supplementary figures and images for: Impact of COVID-19 on Research in Durham University Business School
Source: Sage Open. 2023 Jun 16;13(2):21582440231181314. doi: 10.1177/21582440231181314 (PMC10285187; doi:10.1177/21582440231181314)

Online unpublished Survey instrument


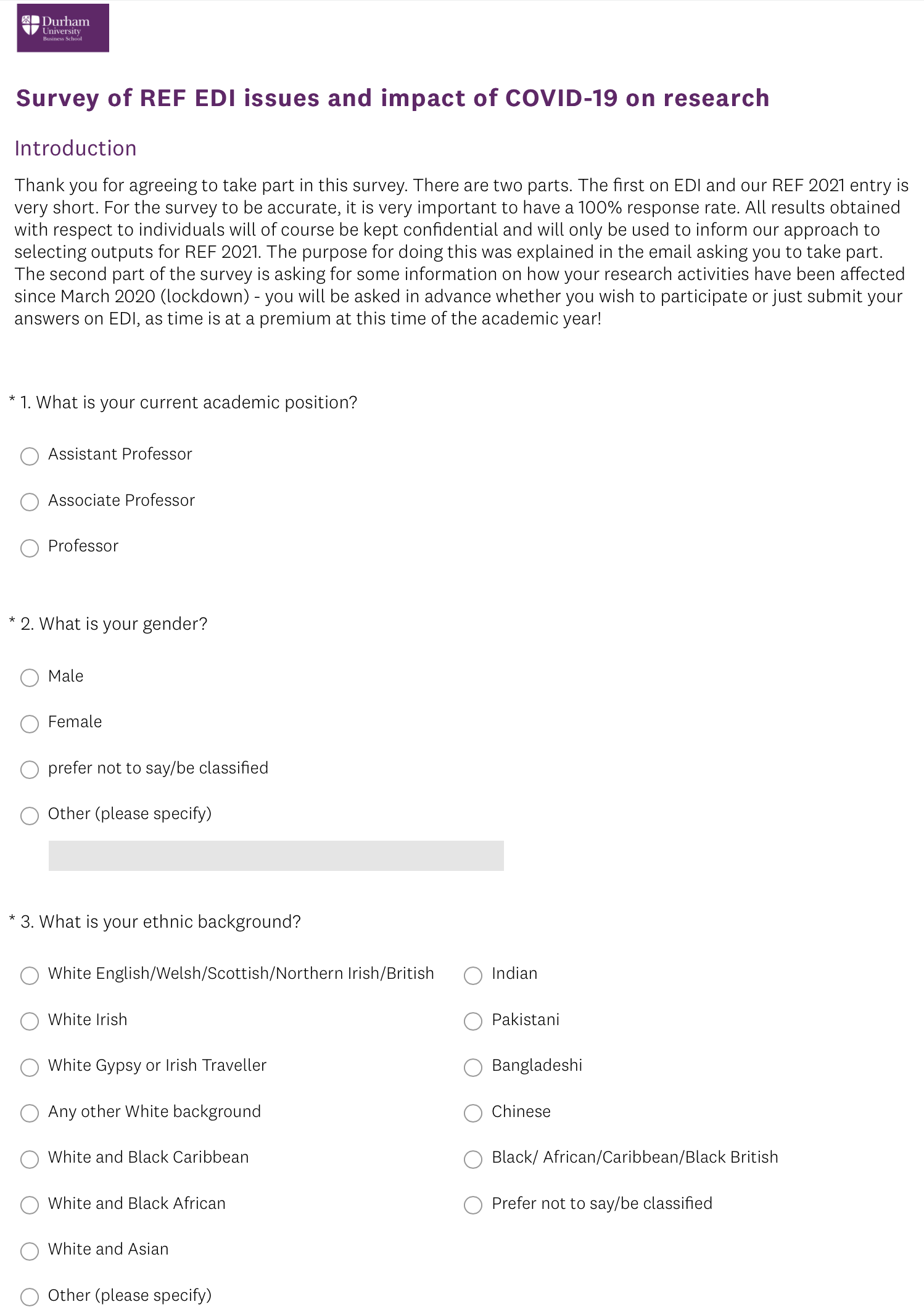


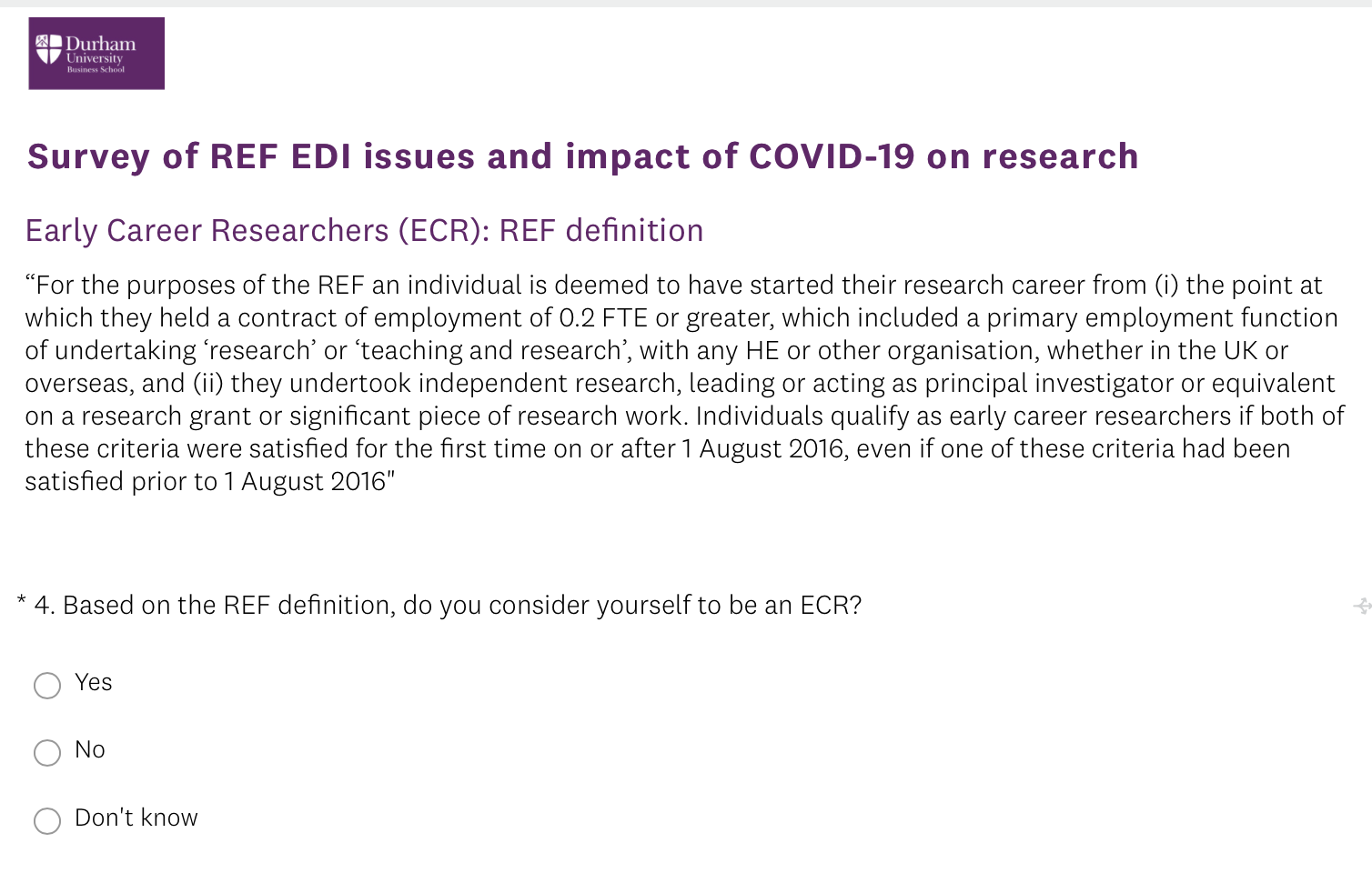


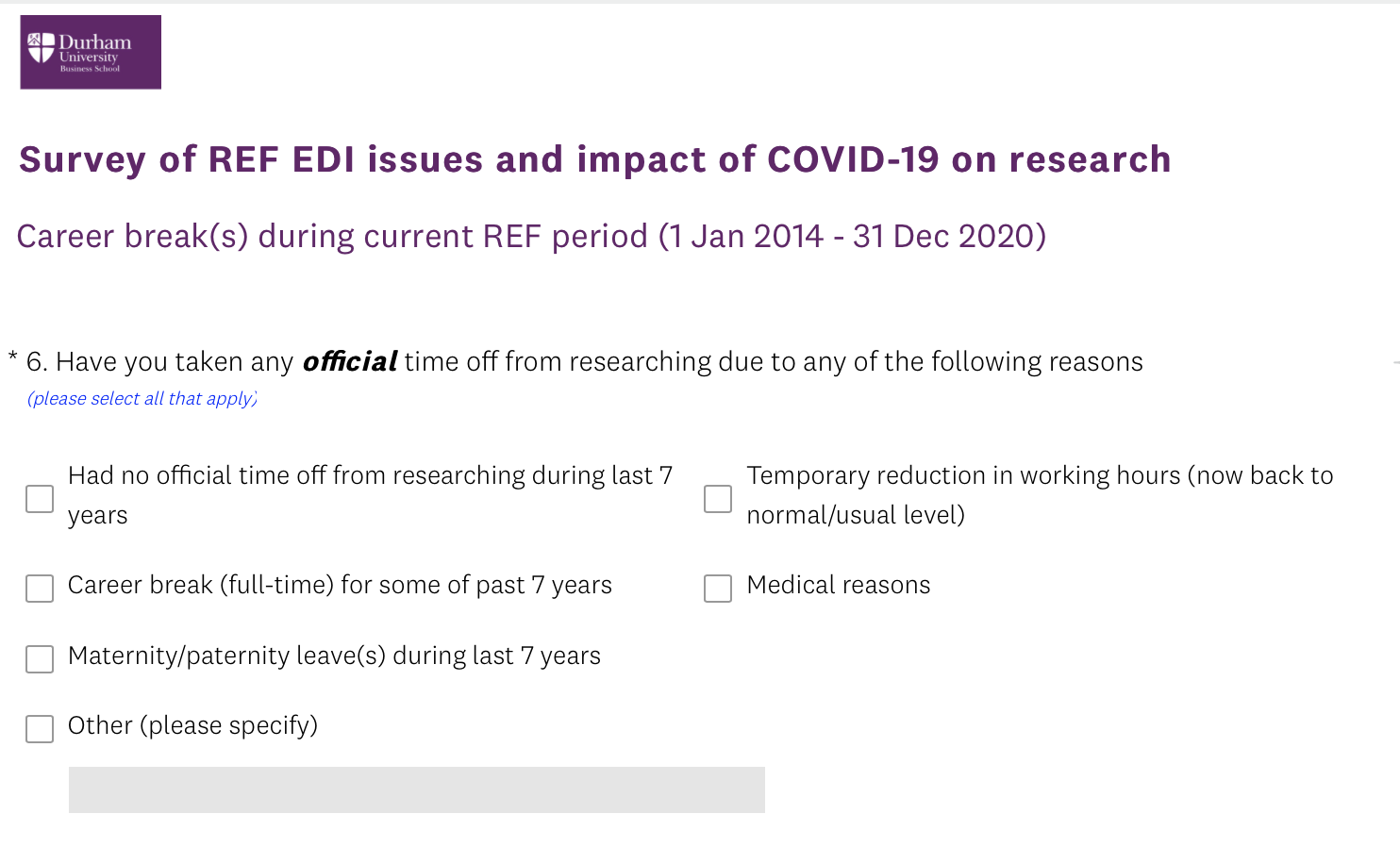

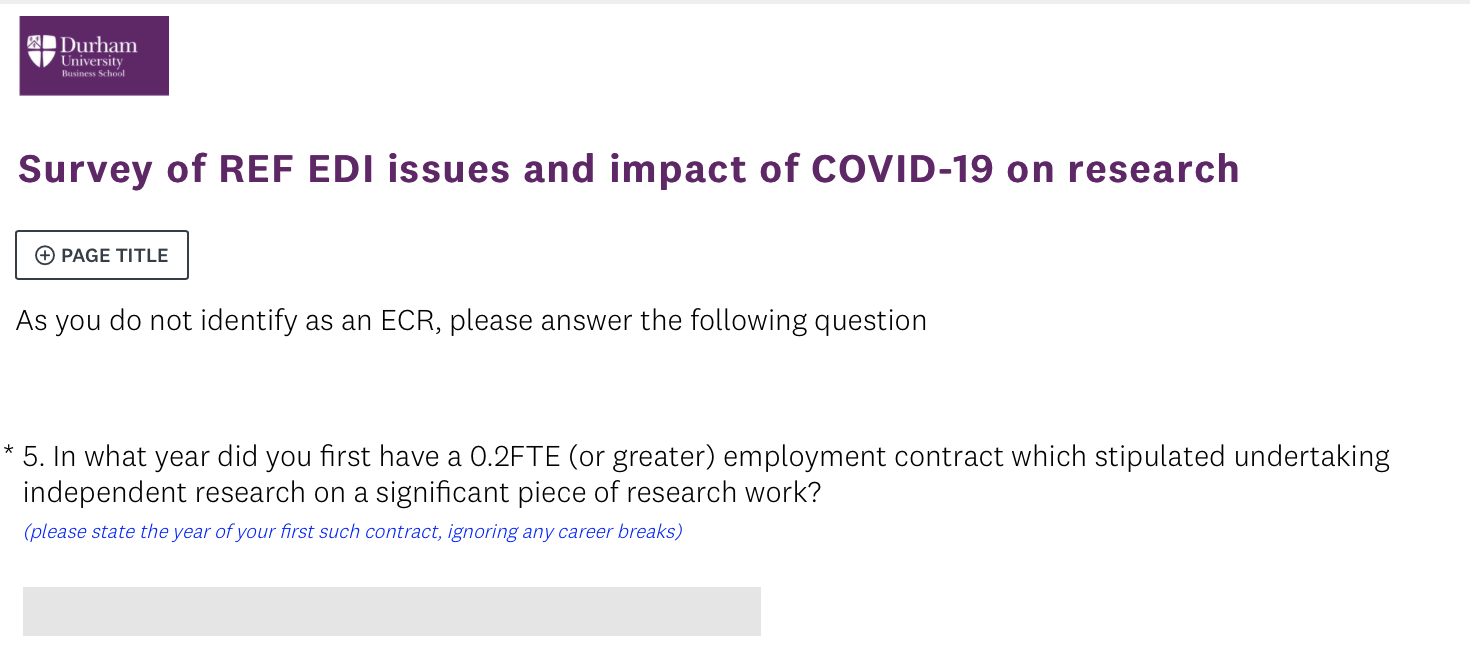


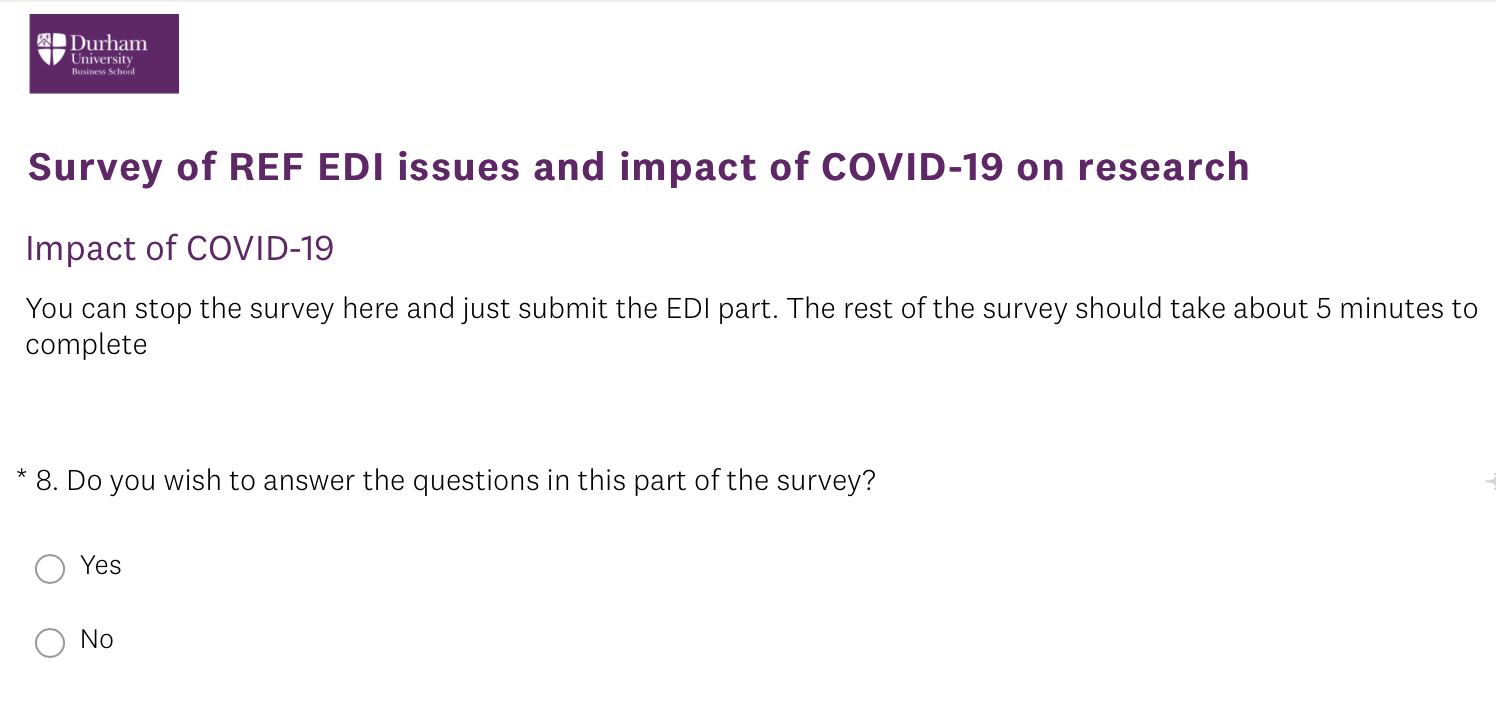

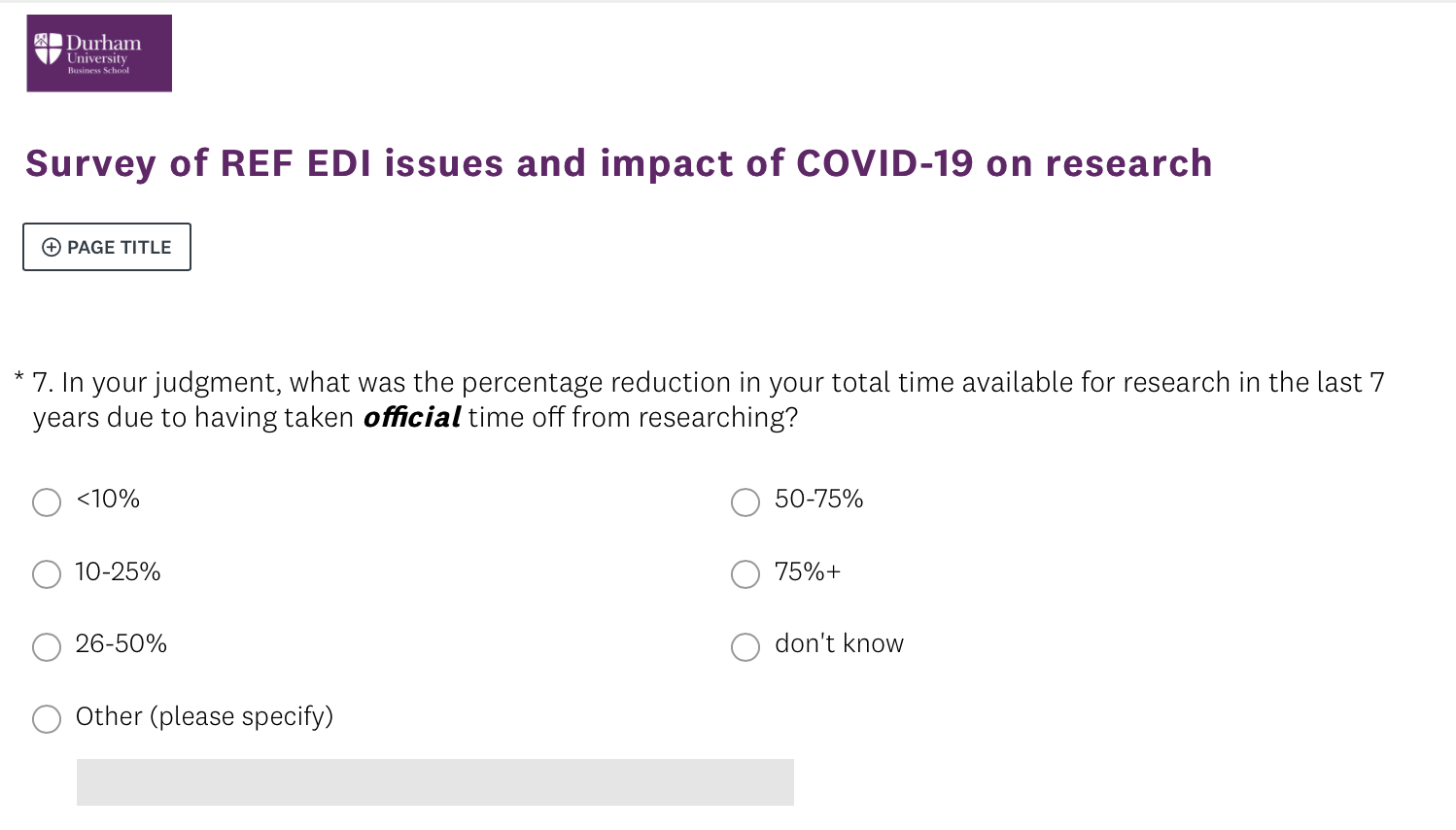

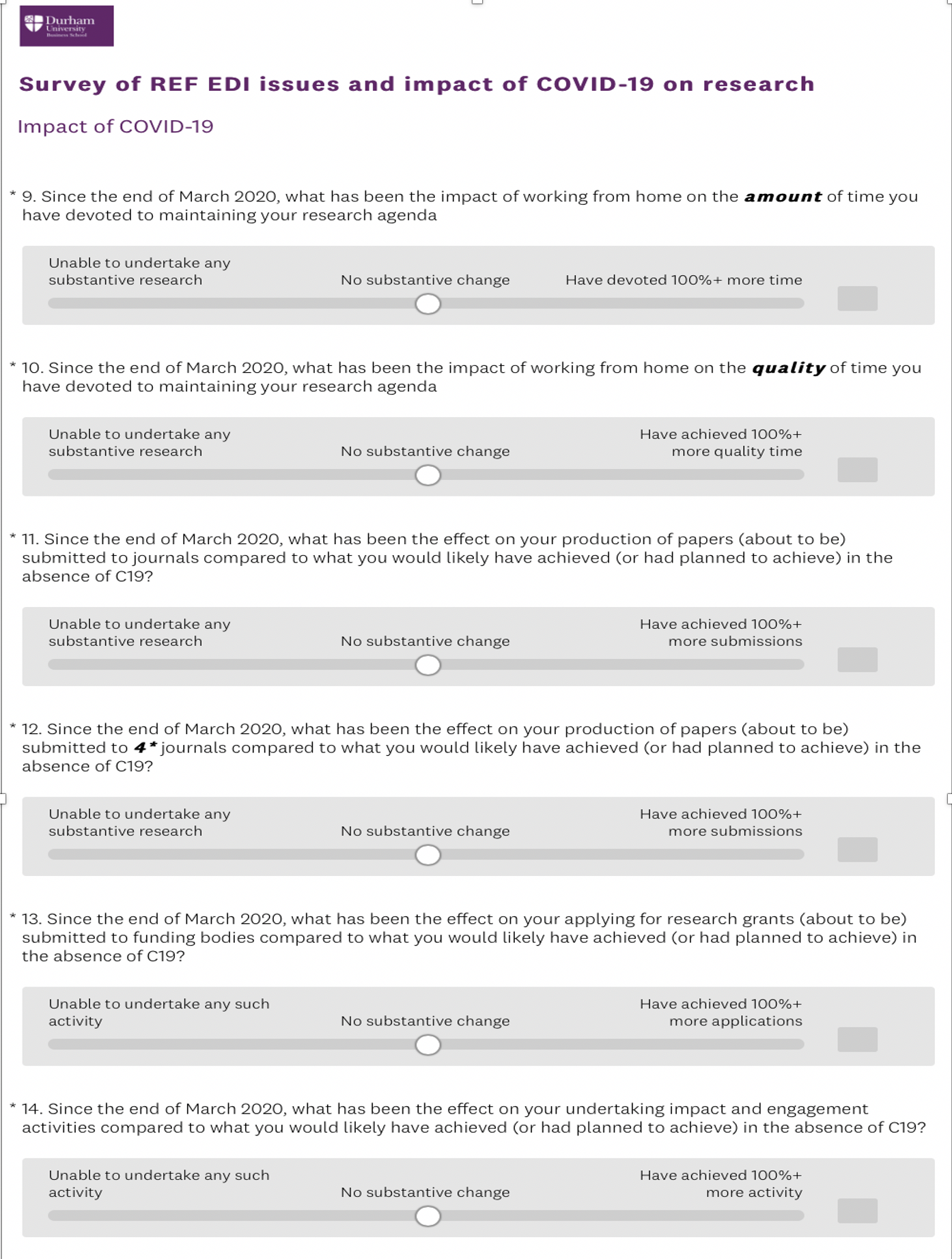

Supplement: sj-docx-1-sgo-10.1177_21582440231181314 – Supplemental material for Impact of COVID-19 on Research in Durham University Business School [file sj-docx-1-sgo-10.1177_21582440231181314.docx]
